# Evaluating the Impact of the COVID-19 Pandemic on First-Year Medical Student Nutrition

**DOI:** 10.7759/cureus.57195

**Published:** 2024-03-29

**Authors:** Chase Yuan, Priya Nair, Christine Jacob, Natalia Lowry

**Affiliations:** 1 College of Medicine, Albany Medical College, Albany, USA; 2 Biochemistry, Albany Medical College, Albany, USA

**Keywords:** calories, stress, medical student, pandemic, covid-19, diet, nutrition

## Abstract

Background: Medical school students in high-stress environments have been shown to make poor personal health decisions ranging from less healthy selections of food to lower rates of exercise. With the emergence of the COVID-19 pandemic adding a major social and health challenge, it is important to examine the effect this new stressor has on the health habits of medical school students.

Methods: All first-year students at Albany Medical College completed a self-recorded three-day log of food intake and exercise modality and duration. Reported data included calories, carbohydrates, protein, lipids, sodium, calcium, and other micronutrients. The data was divided between pre-pandemic (2018-19) and mid-pandemic (2020-21) entries. T-tests and ANOVA tests were used to compare for significance.

Results: Students consumed significantly fewer calories during the pandemic compared to before. This reduction was primarily driven by the female cohort of students. Specifically, this reduction in calories appears to be driven by a decrease in carbohydrate consumption, rather than lipids or protein, which did not change. Additionally, there were significant decreases in sodium, calcium, and vitamin D intake. The mid-pandemic time span (2020-21) appears to be correlated with decreased caloric intake, as well as other important nutrients such as vitamin D and calcium.

Conclusions: This study demonstrated a decrease in calories, carbohydrates, sodium, and alcohol consumption when comparing pre- and mid-pandemic dietary habits of first-year medical students. It also indicated less than the recommended amount of calcium and Vitamin D consumption. Such changes may indicate restrictive eating habits in times of stress.

## Introduction

The relationship between high-stress environments and increases in unhealthy dietary and poor lifestyle habits is well known. Numerous studies have demonstrated the deleterious effects of high stress on dietary choices, ultimately leading to compromised health [1-6]. For instance, Vitamin D is important for bone health, particularly in the female population. Fiber is essential for gut motility and reduces the risk of colorectal cancer. Excessive sodium intake is a well-established cause of hypertension. The association between stress and lifestyle habits has been demonstrated prior to the COVID-19 pandemic and has only intensified with the onset of the pandemic. Even healthcare workers, despite their position as medical providers, are not immune to the effects of stress and poor food choices. In the hospital, physicians working long hours often resort to fast food for sustenance or skip meals altogether due to constraints of time. 

A Canadian study conducted prior to the pandemic found that physicians and on-call residents in an urban teaching hospital reported that inadequate food intake and dehydration had negative impacts on their work performance and sense of well-being [7]. The correlation between stress and nutrition has also been observed in medical school students. The stress of medical school tends to lead to unhealthy or less-healthy nutrition choices. One study in Lithuania examining first- and third-year medical students found that only 20% of students ate the daily suggested 400 grams (g) of fruit and vegetables [8]. In addition, the study found the majority of students engaged in none-to-low amounts of exercise each week, especially among first-year students. A Saudi Arabian study found that academic stress was correlated to feelings of loss of control regarding dietary choices as well as increased soft drink consumption [9]. Bergeron et al. found that while the majority of pharmacy and medical students consumed appropriate sodium and cholesterol, only 10% reported consuming eight or more servings a day of fruit and vegetables [10]. In addition, only 41% of students exercised more than 150 minutes per week. Interestingly, the study found that the major reason students were unable to meet nutritional goals was attributed to lack of time. From such studies, it is clear that many students are not engaging in healthy behaviors, which may lead to health concerns and poor habits later in life. School is a pivotal time for students to not only learn about the science of nutrition but also to develop and implement valuable nutritional habits. Such habits will serve students well in school and beyond by improving cognition and attention, boosting energy, and reducing academic and clinical errors.

The onset of the ongoing SARS-CoV-2 (COVID-19) pandemic brought new challenges to students globally in the medical field. Along with disruptions to in-person classes, the pandemic affected lifestyles, social events, and personal health. The results of studies examining lifestyle changes during the pandemic among students studying health and medicine have been mixed. One study in Vietnam found that 57% of medical and nursing students self-reported no change or unhealthier diet while the remaining 43% reported healthier eating behavior during the pandemic [11]. An American survey of medical students examining personal mood, exercise, sleep, and nutrition revealed a decline in overall wellness during the pandemic [12]. Specifically looking at the lockdown period in Croatia, Dragun et al. found that 32% of students reported weight loss during lockdown and 19% reported weight gain, while physical activity did not change [13].

In order to understand how medical students’ dietary nutrition changed as a result of the COVID-19 pandemic, our study at Albany Medical College compared the nutrition of first-year medical students in the late fall season of 2018 and 2019 (pre-pandemic) to that of first-year students in late fall 2020 and 2021 (mid-pandemic). This study evaluated how medical students’ nutrition changed along with the progression of the COVID-19 pandemic. Understanding these changes could help guide future education programs that attempt to educate and guide medical students to a healthier lifestyle.

## Materials and methods

As a requirement of a nutrition longitudinal education course, all first-year medical students at Albany Medical College (AMC) were required to submit a consecutive three-day food and activity log using the health application MyFitnessPal as part of a consecutive cohort study. The students were instructed to log all meals, snacks, and drinks and submit the records as screenshots with nutrition data to an online portal. The submission also required a written self-reflection of students' dietary and exercise habits. Some logs also contained information on height and weight, but the data was not consistent enough for record entry. Deidentified records from 2018 to 2021 were collected and a retrospective analysis was performed to examine nutrition, caloric intake, and exercise. This data was collected from 445 students from AMC in the late fall to early winter time period of each year. The data was collected through the students’ individual submissions via the online nutrition page. As this was a required assignment for all students, the response rate was 100 percent. All data was numbered and entered into a spreadsheet by the investigators. Descriptive analysis was conducted to identify trends in the data. 

The nutrition data was collected using MyFitnessPal, a health tracker and food nutrition database including macro- and micronutrients. The database includes nutrition from restaurants, pre-prepared meals, and raw ingredients. All reported information was organized by variables that included total calories (calories, Cal), carbohydrates (grams, g), lipids (grams), protein (grams), sodium (milligrams, mg), vitamin D (international units, IU), calcium (milligrams), alcohol (average number of standard drinks), sweetened drinks (average number of standard drinks), exercise (Y/N), and takeout meals (average number of orders). Alcoholic drinks were defined according to standard beverage sizes including 12 fl oz. for beer, 5 fl oz. for wine, or 1.5 fl oz. for distilled spirits. Beverages with added sugar were defined by standard serving sizes of 8 fl oz. and included sweetened fruit juices, soda, and sugared coffee or tea. The total number of drinks consumed was averaged over the three days. Takeout meals were defined by the number of meals bought from a branded restaurant (Chipotle, Dunkin', etc.) and were similarly averaged over three days. Cafeteria food and prepackaged store-bought meals were not included in this selection. Exercise was defined by the presence of any heart rate elevating physical regimen or walking greater than one hour, with "0" meaning no exercise that day or "1" meaning some exercise was completed that day. The exact quantity or quality of exercise was not recorded as it was too inconsistently recorded across student entries. Each student's data entry and written reflection were manually entered into a spreadsheet program.

The criteria for exclusion of incomplete data points were determined by the following: if an entry was missing total calorie data, then the entire entry was excluded. The remaining entries comprised the "qualified responses" category. If an entry missed a specific nutrition category, then the entry was excluded for the specific category, but the rest of the data points for other categories were preserved. This includes "elective" categories such as alcohol, sweet drinks, and takeout meals, as well as exercise. For example, if a student reported alcoholic drink consumption on one day but did not specifically mention alcohol abstinence on the other two days, the two other days were tallied as "0 drinks." Similarly, if a student explicitly mentioned not participating in a behavior, it was written down as "0" for all three days. However, if a student did not report any indication of behavior, their data was omitted from that category, as we could not be sure if the student intentionally omitted the information or merely forgot to report it. Thus, the data was only representative of individuals who at least partially engaged in such behavior and did not include those who made no record at all. Data was analyzed using T-tests comparing pre- and mid-pandemic, and ANOVA for significance comparing the individual years from 2018-2021.

## Results

**Figure 1:**
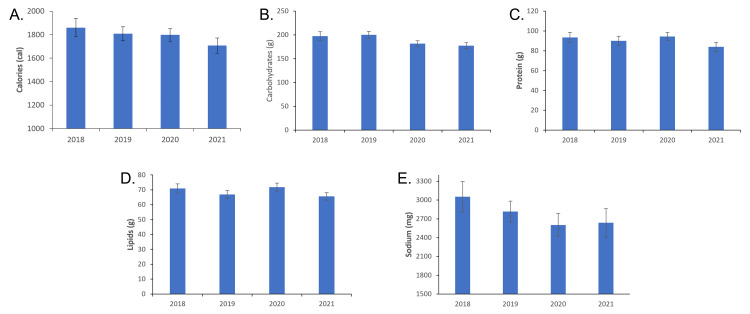
Major nutrition markers (2018-2021). ANOVA test of significance did not yield any significant change between any year. A: Average daily caloric intake by year. B: Average daily carbohydrate intake by year. C: Average daily protein intake by year. D: Average daily lipid intake by year. E: Average daily sodium intake by year.

From 2018 to 2021, a total number of 445 students answered the survey. Of these, 120 responses were disqualified due to missing records of total caloric intake or otherwise incomplete nutrition logs. Of the remaining 325 students, 59 students were from 2018, 90 from 2019, 91 from 2020, and 85 from 2021. ANOVA analysis of total calories, carbohydrates, protein, lipids, or sodium intake revealed no significant change across all four years (Figure 1).

However, comparing pre-pandemic vs mid-pandemic yielded some distinction. There was a significant decrease in overall caloric intake from 1829 Cal ± 43 to 1754 Cal ± 43 (p ≤0.05) when comparing pre-pandemic (2018-19) and mid-pandemic (2020-21) diets respectively (Table 1). This represents a 4% decrease in caloric intake. Both pre- and mid-pandemic caloric consumption is below the recommended daily value of 2000 calories [14].

**Table 1:** Average caloric, macronutrient, micronutrient, elective food, and exercise quantities. Pre-pandemic (2018-2019) and mid-pandemic (2020-2021) categories of health logs were compiled and averaged. The averages for each category represent daily intake or participation in an activity such as ordering takeout or exercising. For elective food (alcohol, sweet drinks, takeout meals) and exercise, these categories were only averaged among those who participated in the activity. Those who did not engage in such behaviors were excluded from calculations. * = p<0.05, ** = p<0.01.

	2018-2019 (n=149)	2020-2021 (n=176)
Combined total calories (Cal)*	1829±42	1754±43
Male total calories (Cal)	2028±47	2025±51
Female total calories (Cal)**	1581±37	1496 ±27
Carbohydrates (g)**	203±10	179±5
Proteins (g)	91 ±3.6	90±3.5
Lipids (g)	69±2.4	69±2.4
Sodium (mg)*	2917±114	2621±101
Calcium (mg)*	804±35	734±32
Vitamin D (IU)**	316±55	202±24
Alcohol (standard drinks/day)**	0.27±0.07	0.11±0.03
Sweetened drinks (standard drinks/day)	0.29±0.04	0.35±0.05
Takeout meals (number/day)	0.6±0.07	0.5±0.04
Physical activity (average number of active days)	0.56±0.04	0.57±0.04

**Figure 2:**
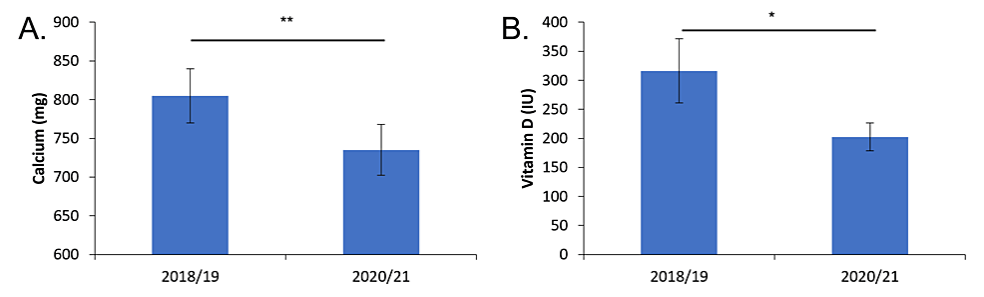
Calcium and vitamin D intake pre- and mid-pandemic. A: Average daily calcium intake. B: Average daily vitamin D intake. * = p<0.05, ** = p<0.01.

This data was further categorized by males vs females. For males, there was no change in calorie consumption when comparing 2018-19 to 2020-21 (2028 Cal ± 47 vs. 2025 Cal ± 51, Figure 2, Table 1). For females, there was a significant decrease in calorie consumption between 2018-19 and 2020-21 (1581 Cal ± 37 vs. 1496 Cal ± 27, p<0.01, Figure 2, Table 1).

When examining carbohydrate intake, there was a significant decrease in carbohydrate consumption from 203 g ± 10 to 179 g ± 5, (p ≤0.01) when comparing pre- and mid-pandemic diets respectively (Figure 2). This represents a 13% intake decrease. Both pre- and mid-pandemic carbohydrate consumption is below the recommended daily value of 275 g of carbohydrates [14]. There was not a significant change in the consumption of lipids or protein across both two-year time frames. However, consumption of lipids pre- and mid-pandemic was below the recommended daily value of 78 g.

**Figure 3:**
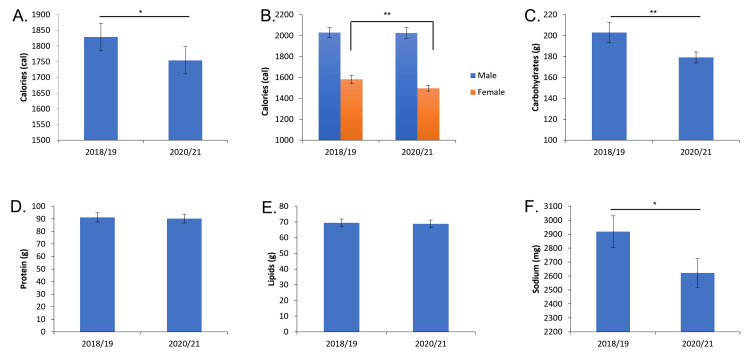
Major nutrition markers intake pre- and mid-pandemic. A: Average daily caloric intake. B: Average daily total caloric intake as differentiated by sex. C: Average daily carbohydrate intake. D: Average daily lipid intake. E: Average daily protein intake. F: Average daily sodium intake. * = p<0.05, ** = p<0.01.

Our research revealed a statistically significant decrease in both calcium and vitamin D intake from 2018-19 compared to 2020-21 (Figure 3). Average calcium intake decreased from 804 mg ± 35 to 735 mg ± 33 (p ≤0.05). Average vitamin D consumption decreased from 316 IU ± 55 to 202 IU ± 24 (p ≤0.01). Both of these values are below the recommended daily value of 600 IU [14].

**Figure 4:**
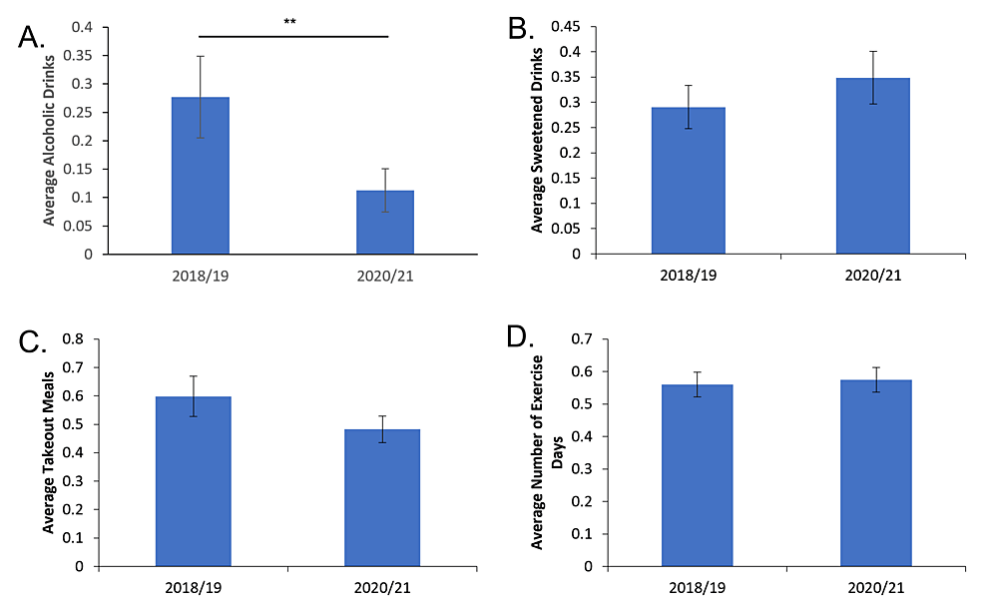
Average daily sweet drinks, takeout meals, alcoholic drinks, and exercise over a three-day period among those who engaged in elective behavior. A: Average daily number of alcoholic drinks intake over the recorded three-day period. B: Average daily number of sweetened beverages over the recorded three-day period. C: Average daily number of takeout meals over the recorded three-day period. D: Average number of days with exercise. ** = p<0.01.

Among the individuals who consumed sweetened beverages, there was a slight increase trend in the daily average consumption from pre-pandemic to mid-pandemic (0.29 avg drinks ± 0.04 vs 0.34 avg drinks ± 0.05); however, it did not reach statistical significance (Figure 4, Table 1). When examining alcohol consumption, daily use of alcohol significantly decreased when comparing pre- and mid-pandemic consumption (0.27 avg drinks ±0.07 vs 0.11 avg drinks ± 0.03, Figure 4, Table 1). Takeout meal consumption did not meaningfully change from before to during the pandemic (0.6 avg meals ± 0.07 vs 0.48 avg meals ± 0.04 respectively, Figure 4, Table 1).

## Discussion

Interpreting changes in nutrition and exercise

This study has demonstrated lifestyle changes in medical students in light of the COVID-19 pandemic. The diets of first-year medical students (MS1s) at Albany Medical College between 2018-19 were noted and compared to the diets of MS1s between 2020 and 2021. Results showed significant changes in overall caloric intake, and consumption of carbohydrates, sodium, and alcohol, but no significant changes in fat and protein intake.

Changes in calories and macronutrients

Prior studies such as Coleman et al. have demonstrated that medical students do not meet the recommended daily food intake value of 1600-2400 calories for women, and 2000-3000 calories for men [14,15]. Of the 193 students who completed the Coleman survey, students on average were meeting 93.4% of the daily value. This is partially consistent with the results of our study, which found that students were on average meeting 96% of the recommended daily intake (RDI) of 2000 calories. Women had a significant decrease in average caloric intake from 1829 calories in 2018-19 to 1754 calories in 2020-21. However, the caloric intake of men remained at recommended levels pre- and mid-pandemic, slightly decreasing from 2028 to 2024 calories respectively. This suggests the pandemic may exacerbate certain nutritional deficiencies, particularly in female medical students.

The decrease in calories is reflected by a reduction in carbohydrate intake. Carbohydrates, which provide 4 Cal/gram, are the major contributor to caloric consumption. Protein and lipid intake did not significantly differ before and during the pandemic. This is consistent with another study that found the stress from the lockdown led to a greater likelihood of restrictive eating in college-eating students [16]. It is also possible that mild eating disorders were exacerbated by the onset of the pandemic [17].

Decreases in sodium intake

The significant decrease in sodium has several possible explanations. First, it may be decreased in correlation to the decrease in carbohydrates consumed, as carbohydrate-rich foods are often also sodium-rich. Second, it may be that students are increasing their health consciousness; the pandemic has stressed the importance of staying healthy, and reducing sodium intake is a beneficial start to this process. In any case, our findings are consistent with the findings from Bergeron et al. in which 73% of medical and pharmacy students who completed their questionnaire had a sodium intake less than the RDI of 2300 mg/day [10]. It is important to note that intentional sodium restriction can lead to hemodynamic complications, particularly in those who already may demonstrate eating disorders or have stress-induced habits that result in reduced food intake [18].

Decreases in calcium and vitamin D intake

Vitamin D plays a critical role in the wellness of all age groups. Vitamin D is produced in the skin and is predominantly found in animal food sources. Additionally, it is often supplemented in diet through capsules or artificially added to food. Vitamin D undergoes hydroxylation within the liver and kidney to become active vitamin D3, calcitriol, or 1,25-dihydroxycholecalciferol. This vitamin D3 is required for calcium absorption, maintenance of calcium and phosphate in the blood, inflammation reduction, and glucose metabolism [19].

Calcium is important in the maintenance of bone strength, muscle movement, cell-cell communication, signal transduction, and protein function. It is in part regulated by the presence of active vitamin D, which increases absorption of calcium in the gut. Low intake of calcium triggers the body to dissolve bone, releasing stored calcium. Over time, this reduces the strength of bone, leading to microfractures and deformation. Both vitamin D and calcium are critical in maintaining health in an individual.

Our study showed significant decreases in intake of both calcium and vitamin D. We found a significant decrease in calcium consumption for MS1 students in 2018-19 from 804 mg to 734 mg. These findings are congruent with the Coleman et al. study, which demonstrated that students were already failing to meet the daily recommended value of either micronutrient prior to the COVID-19 pandemic [15]. While the RDI of calcium is 1300 mg, the students in the Coleman study had an intake of 899 mg. Furthermore, while the RDI of vitamin D is 600 international units or 15 mg, these students had an intake of 5.7 mg. Therefore, Coleman’s study emphasizes that students prior to the pandemic were already falling short of meeting the RDI. According to our data, the problem has worsened following the pandemic. The results of our study, as well as Coleman’s study, are also supported by those of a study by Al-Elq et al., which analyzed the Vitamin D levels in the blood of 198 medical students in the pre-clerkship years of a Saudi medical school [20]. This study found that 100% of their students had low Vitamin D levels. More specifically, 96% were vitamin D deficient, while 4% were vitamin D insufficient. In the northern latitudes of Albany, New York where sunlight is limited during the winter months, it is even more important to maintain sufficient vitamin D levels to maintain bone integrity and prevent bone loss.

Decreases in alcohol intake

When examining alcohol consumption in first-year medical students before and during the pandemic, our study demonstrated a significant two-fold decrease in alcohol consumption. These results align with a study of science students in Spain by Rodríguez-Pérez et al., who were found to have a decrease in overall alcohol intake following the pandemic [21]. That study demonstrated that of the 4122 students who followed a Mediterranean diet plan, 50% showed a decrease in alcohol intake, 10.9% an increase, and 39.1% no change. This could reveal a culture that places a high emphasis on social dependence when it comes to alcohol consumption; with fewer social occasions, students consequently drank less alcohol. The results of our study were further consistent with previous reports on college students whose consumption also decreased during the pandemic [22].

Sweet beverages

Previous studies have found mixed results pertaining to the use of students’ sweetened drink consumption during the pandemic. Specifically, Jia et al. found a notable increase in sweetened drink consumption among high school and undergraduate students, but a decrease among graduate students [23]. This is similar to Mazza et al. who found that Italian university students consumed more sweetened drinks since the pandemic [24]. However, a study in South Korea by Kim et al. found that adolescents consumed fewer sweetened beverages [25]. Our findings, which indicate no significant change in consumption, suggest that such habits have not been influenced by the pandemic. It is possible that sweet drink consumption is correlated to social gatherings as similar to alcohol use, but also that time spent at home promotes drinking sugary beverages as relaxation without the inebriation associated with alcohol. Furthermore, beverages such as energy drinks may be used as stimulants for students while studying.

Exercise

Finally, regarding exercise, our results are inconsistent with other studies. The study by Bergeron et al. showed an increase in sedentary activity in students pre- and mid-pandemic [10]. That study showed a three-fold increase in the amount of time spent on a computer or tablet. Furthermore, a study by Husain & Ashkanani looked at 415 randomly selected adults living in Kuwait, ranging from the ages of 18 and 73 [26]. This study found a significant increase (16.1% to 43.6%) in the number of individuals who spent over 6 hours a day on electronic devices. In contrast, our study found no significant difference between pre- and mid-pandemic rates of exercise. According to anecdotal testimonies in the written reflection portion of the nutrition assignment, some students described increased consciousness toward health while others described mental fatigue discouraging them from committing to exercise. As our study did not demonstrate any significant change, further investigation would be needed to study this consequence of remote learning caused by the pandemic.

Limitations and future directions

One drawback of this study is that a self-reported three-day food and exercise log submitted online is insufficient to fully capture the long-term health habits of students, particularly as the exact three days recorded are not standardized to the same set of days of the week. As such, indulgences such as alcohol may be overrepresented in those who recorded weekend logs compared to weekday logs. Furthermore, many students did not report the presence or absence of exercise, which is important to understand daily caloric requirements. This drawback may be mitigated with a sufficiently large sample size to cover all possible three-day intervals, or alternatively, a one-week study may be more thorough. Furthermore, narrowing the timeframe to record data would also improve consistency, since in this study, students were given between October and December to complete this assignment.

An additional limitation is the number of variables collected. It would be beneficial to record more data such as additional micronutrients, fiber, quantity, and quality of exercise, and more individual details such as the body-mass index to estimate individual caloric needs rather than assume a 2000-calorie diet for everybody. It would also be interesting to follow the same cohort before and during the pandemic. However, that method would also be confounded by the transition through medical school and the associated change in environmental stressors. One further limitation is that not all students reported each variable. For instance, some students neither affirmed nor denied alcohol consumption. For those lacking such data points, there was no choice but to exclude those entries from our analysis. Ideally, each student reports on each variable, even if they did not engage in such behavior.

Finally, it would be beneficial to gather the students' anecdotal thoughts on the pandemic and how it relates to the students' dietary habits. This would provide a new perspective on the link between changes to diet and the pandemic itself.

## Conclusions

This study indicates that there were significant changes between pre- and mid-pandemic dietary habits of first-year medical students at Albany Medical College. Through the decrease in calories, carbohydrates, and sodium, there may be a trend toward restrictive eating habits in times of stress. In addition, it appears that many students have low levels of vitamin D and calcium intake. As both are important to maintain homeostasis, it is imperative that greater education for the medical students be placed on nutrition. Finally, the decrease in alcohol consumption indicates a possible social component to this change. However, more data needs to be collected in order to make a definitive conclusion. Some of the limitations of our study included the relatively small sample size and duration of the study. Although the sample size is limited by medical class size, further studies can investigate if the behaviors and nutritional habits that developed during the pandemic will begin to revert as the pandemic subsides or if there will be permanent changes to students' lifestyles.
